# Identification of Maturity-Onset Diabetes of the Young Caused by Mutation in *FOXM1 via* Whole-Exome Sequencing in Northern China

**DOI:** 10.3389/fendo.2020.534362

**Published:** 2021-02-09

**Authors:** Liang Zhong, Zengyi Zhao, Qingshan Hu, Yang Li, Weili Zhao, Chuang Li, Yunqiang Xu, Ruijuan Rong, Jing Zhang, Zifeng Zhang, Nan Li, Zanchao Liu

**Affiliations:** ^1^ The Shijiazhuang Second Hospital, Shijiazhuang, China; ^2^ Hebei Provincial Key Laboratory of Basic Medicine for Diabetes, The Shijiazhuang Second Hospital, Shijiazhuang, China; ^3^ Shijiazhuang Technology Innovation Center of Precision Medicine for Diabetes, The Shijiazhuang Second Hospital, Shijiazhuang, China

**Keywords:** maturity-onset diabetes of the young (MODY), diabetes, whole-exome sequencing (WES), SNP, FoxM1

## Abstract

Diabetes mellitus is a highly heterogeneous disorder encompassing different types with particular clinical manifestations, while maturity-onset diabetes of the young (MODY) is an early-onset monogenenic diabetes. Most genetic predisposition of MODY has been identified in European and American populations. A large number of Chinese individuals are misdiagnosed due to defects of unknown genes. In this study, we analyzed the genetic and clinical characteristics of the Northern China. A total of 200 diabetic patients, including 10 suspected MODY subjects, were enrolled, and the mutational analysis of monogenic genes was performed by whole-exome sequencing and confirmed by familial information and Sanger sequencing. We found that clinical features and genetic characteristics have varied widely between MODY and other diabetic subjects in Northern China. *FOXM1*, a key molecule in the proliferation of pancreatic β-cells, has a rare mutation rs535471991, which leads to instability within the phosphorylated domain that impairs its function. Our findings indicate that *FOXM1* may play a critical role in MODY, which could reduce the misdiagnose rate and provide promising therapy for MODY patients.

## Introduction

Maturity-onset diabetes of the young (MODY) is a kind of monogenic diabetes mellitus that is characterized by early-onset, autosomal dominant, non-insulin dependent diabetes. Pancreatic β-cell dysfunction reduces glucose-stimulated insulin secretion during early age due to monogenic variation (1, 2). However, MODY not only manifests a distinct clinical phenotype but also emerges metabolically and genetically heterogeneously due to the various MODY-associated genes. To date, 14 genes (*HNF4A, GCK*, *HNF1A, PDX1, TCF2, NEUROD1, KLF11, CEL, PAX4, INS, BLK, ABCC8, KCNJ11, and APPL1*) have been identified, and their mutations are responsible for the initiation of MODY (3–5). Despite previous intensive linkage analyses for MODY, there are still diagnosed cases that remain genetically inexplicable (6). In addition, different studies suggest that the prevalence of specific mutations of MODY genes differs considerably among various ethnic groups (7). Without characteristic features and pedigreed awareness, most MODY patients are misdiagnosed with type 1 or type 2 diabetes in Chinese populations, who may potentially receive inappropriate therapy.

The emergence of next-generation sequencing has greatly enhanced the identification of novel mutated genes related to complicated diseases. In particular, whole-exome sequencing (WES) is a more useful and efficient strategy for identifying unknown causative genes in complex disorders, such as GCK-MODY (8) PAX4-MODY (9), and KCNJ11-MODY (10). According to Bonnefond’s finding, WES also provides a clinical tool to assess patients presenting with other monogenic diabetes (6). With regard to MODY, geography and ethnicity specific detection rates have been determined in previous studies (11). Moreover, the low detection rate of given mutations previously reported in Chinese patients suggests that the MODY-X gene may play a major role in these populations (12–14).

The aim of this study was to investigate the prevalence of the diabetic population and novel mutations responsible for MODY, especially in Northern of China. Ten diagnosed and suspected MODY patients underwent WES analysis to elucidate the molecular genotype. Focusing on monogenic diabetes enhances the understanding of pancreatic β-cell dysfunction and insulin resistance, which will promote the criterion of clinical typing and reduce misdiagnosis, finally leading to precise and effective therapy.

## Materials and Methods

### Experimental Subjects

Diabetes subjects were recruited from the Bio-resource Center of The Shijiazhuang Second Hospital (Shijiazhuang, China). The suspected clinical diagnoses of MODY patients were selected based on (1) the early onset of diabetes (< 25 years of age); (2) negative pancreatic autoantibodies; (3) persistently detectable C-peptide; (4) nonketotic hyperglycaemia; and (5) non-pedigreed information. The study was approved by the ethical committee of the The Shijiazhuang Second Hospital and all the patients provided their written informed consent to participate in this study.

### Whole-Exome Sequencing

Five to 10 ml venous blood was collected in plastic EDTA bottles or >5 µg DNA. DNA extraction was performed using the Gentra Puregene Blood Kit (Qiagen) according to the manufacturer’s instructions. DNA was quantified for each sample using the Nanodrop (Thermo Fisher Scientific). Whole-exome libraries were constructed using the TruSeq Exome Library Preparation Kit (Illumina, CA). Sequencing was performed using the XTen system (Illumina, San Diego, CA, USA) to generate 2×150 bp paired-end reads. The depth of each sample was over 100X.

### Variant Calling

Sequenced raw reads were mapped against the human reference genome (GRCh38) with Burrows-Wheeler Aligner (BWA, v0.78) (15). Variant identification was performed with Genome Analysis Toolkit (GATK, v4.1.2.0) (16). Duplicate alignments were marked and removed with Picard tool (v2.2). Variant quality filters were applied by a set of criteria (QUAL > 30, 5 < DP) and parameters as recommended by GATK (17). The annotation of variants was performed and ANNOVAR (18).

### Genome-Wide Association Analysis

Variation and phenotype data were analyzed by PLINK (v1.9) (19). The variants with a minor allele frequency (MAF) of less than 0.05, missing call frequencies greater than 0.1 and Hardy-Weinberg equilibrium exact test p-value less than 0.00001 were excluded. The three diabetic groups, MODY group used as case and the T1DM and T2DM used as control, C-peptide and FPG, were selected as phenotypes to perform GWAS (Genome Wide Association Analysis) analysis.

### Gene-Based Rare Variant Association Tests

To test differences burden of the MODY and MODY-X genes, total 56 genes used for burden test were selected by their potential pathogenicity in MODY or the dysfunction of pancreas. RVTESTS was used for the gene-based association by combined multivariate and collapsing (CMC) method (20). Common variants were removed with the following criteria: MAF more than 0.005 in any public database (ExAC, GnomAD, 1000 Genomes) or more than 0.01 in samples of this study; call rate of less than 0.9 in the study samples. The significance threshold here was set to 0.0167 (comparisons between the 3 groups, 0.0167 = 0.05/3), in accordance with Bonferroni correction.

### Statistics

Statistical analysis was conducted by R and related packages. Wilcoxon test was used for pairwise comparison of quantitative traits, and Fisher’s exact test was used for qualitative traits. Kruskal-Wallis H test was used for one-way analysis of variance among groups. The p-value was adjusted by Benjamini & Hochberg method, which is also known as false discovery rate (FDR) (21).

## Results

### Clinical Characteristics of Diabetes Subjects

**Figure 1:**
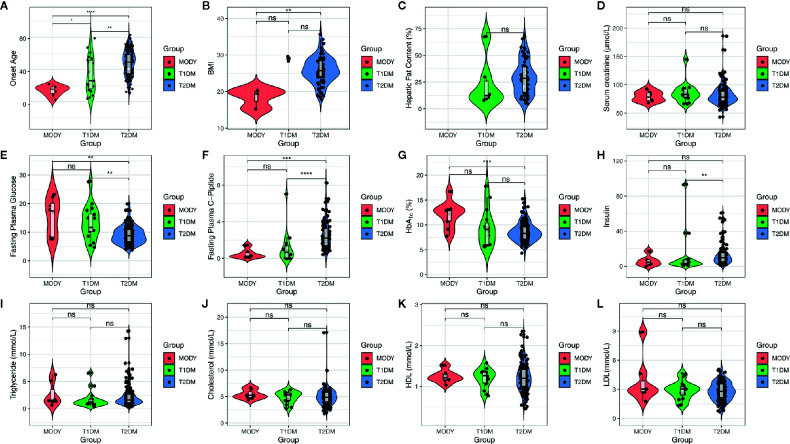
Clinical characteristics of subjects. X axis indicates the clinical characteristics of subjects, which including age, BMI, hepatic fat content, serum creatinine, FPG, FPGC-piptide, HbA_1_ *c*, insulin, triglyceride, cholesterol, HDL, and LDL. And the colors of figure represent three diabetic group, MODY (red), T1DM (green), and T2DM (blue), respectively. The box plot is inside violin plot and the asterisk represents the significance between pairwised comparison. ns, p > 0.05; *p <= 0.05; **p <= 0.01; ***p <= 0.001; ****p <= 0.0001.

We sequenced a total of 200 diagnosed diabetes subjects in this study and found significant differences among the 3 groups (**Figure 1**), such as age (*P* = 2.60 × 10^-8^, Kruskal-Wallis test) fasting plasma glucose (FPG) (*P* = 0.00028, Kruskal-Wallis test), fasting plasma C-peptide (FPGC-peptide) (*P* = 2.80 × 10^-7^, Kruskal-Wallis test), insulin (*P* = 0.0023, Kruskal-Wallis test), HbA_1_ *c* (*P* = 0.0015, Kruskal-Wallis test), and body mass index (BMI) (*P* = 0.0079, Kruskal-Wallis test). There were 130 males (65%) and 70 females (35%), and the average physical age was 53.67 ± 16.83, which ranged from 2 to 87 years. We unexpectedly found significant differences between the suspected MODY group and T1DM or T2DM group within onset age; however, we found that there were significant differences in BMI (*P* = 0.023, Wilcoxon test), HbA_1_ *c* (*P* = 0.00072, Wilcoxon test), FPGC-peptide (*P* = 0.00044, Wilcoxon test), and FPG (*P* = 0.017, Wilcoxon test) between the suspected MODY group and T2DM group, whereas those results did not show a remarkable alteration within the MODY group and T2DM group. Comparatively, the T1DM group also differed from T2DM group in FPGC-peptide (*P* = 3.90 × 10^-5^, Wilcoxon test), FPG (*P* = 0.006, Wilcoxon test), and insulin (*P* = 0.0037, Wilcoxon test) (**Table 1**). These finding suggested that a number of clinical characteristics have varied widely among various diabetic subjects.

**Table 1:** Clinical characteristics of diabetes subjects.

	MODY	T1DM	T2DM	P-value	Significance
**Sex(female male)**	0\10	12\10	58\110	0.011	*
**Age**	22.6 ± 15.04	41.86 ± 18.75	57.07 ± 13.9	2.60E-08	****
**FPG**	15.49 ± 6.57	13.4 ± 5.88	9 ± 3.01	0.00028	***
**Insulin**	6.22 ± 6.11	12.15 ± 24.23	12.83 ± 11.75	0.0023	**
**FPGC-peptide**	0.49 ± 0.51	1.13 ± 1.7	2.5 ± 1.46	2.80E-07	****
**HbA_1_*c***	12.12 ± 2.59	9.41 ± 3.7	8.59 ± 1.9	0.0015	**
**Hepatic Fat Content**	NA	23.45 ± 23.16	29.09 ± 15.55	0.2	ns
**Triglyceride**	2.63 ± 2.18	1.72 ± 1.59	2.24 ± 1.94	0.15	ns
**Cholesterol**	5.42 ± 0.86	4.87 ± 1.08	4.95 ± 1.54	0.48	ns
**HDL**	1.24 ± 0.15	1.21 ± 0.23	1.22 ± 0.32	0.92	ns
**LDL**	3.85 ± 2.39	2.89 ± 0.91	2.82 ± 0.91	0.57	ns
**Serum creatinine**	79.34 ± 8.83	86.01 ± 18.91	81.2 ± 17.06	0.5	ns
**BMI**	18.38 ± 2.71	29.06 ± 0.93	25.76 ± 3.49	0.0079	**

^1^The one-way analysis of variance was performed by the Kruskal–Wallis test. The P value was adjusted by the Benjamini & Hochberg (FDR) method.

^2^ns, p > 0.05; *p <= 0.05; **p <= 0.01; ***p <= 0.001; ****p <= 0.0001.

### Identification and Annotation of Variations

**Figure 2:**
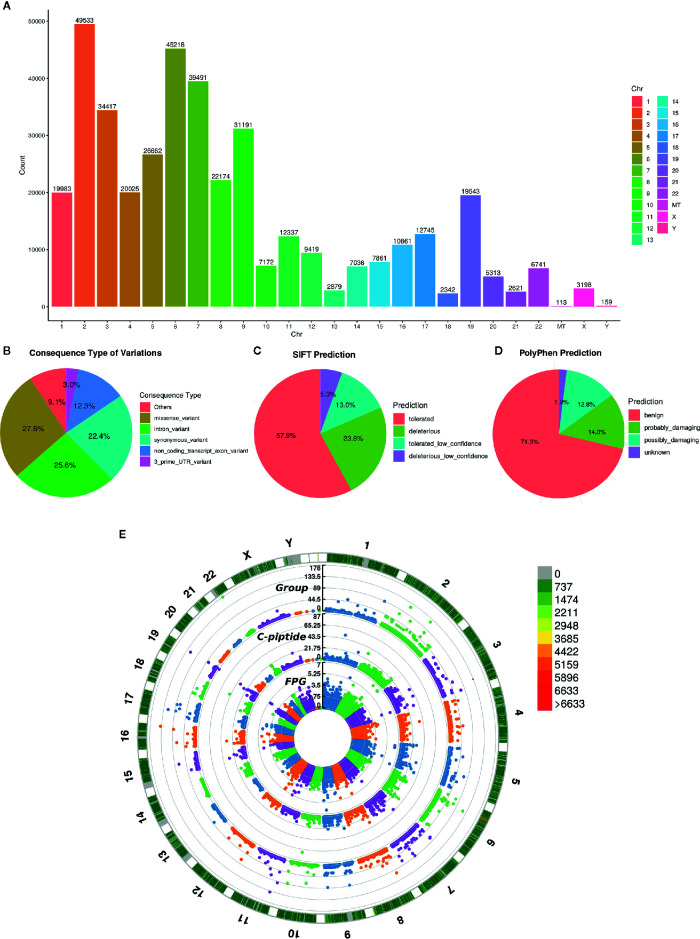
Variant annotation and GWAS analysis. **(A)** The count of mutation distributed in each chromosome, the X axis indicates the name of chromosome and the Y axis is the count of mutation. **(B)** Consequence type of identified variations, top five categories of variations are listed. **(C, D)** The pie chart of pathogenic prediction, variations annotated by Sift and PolyPhen2. **(E)** Manhattan plot of GWAS results, the most outside track indicates the density of mutation, colors represent the count. The 3 tracks of manhattan plot are the GWAS results calculated by diabetic group, C-piptide and FPG, respectively. The vertical axis of 3 tracks represents the -Log10(P-value).

To investigate the relationship between genetic polymorphism and different types of diabetes, we performed whole-exome sequencing of the 200 diabetic cases, including T1DM, T2DM and MODY. The depth of WES achieved >100× coverage for all samples. The joint variants distributed in the genome shown in **Figure 2A** and the consequence type of variants are displayed in **Figure 2B**, including total 1,941,129 variants called from WES data, of which 417,468 remained after stringent filtering criteria were applied. We used SIFT (22) and PolyPhen2 (23) to predict whether an amino acid substitution could have an impact on the biological function of proteins, which showed that 23.8 and 26.82% variants might have a deleterious effect on the protein function (**Figures 2C, D** **)**. However, according to ACMG guidelines, we found only eight likely pathogenic variants and six pathogenic variants that annotated by InterVar (24), unfortunately, none of those 14 variants was associated with diabetes.

Then, principal component analysis (PCA) was conducted on the genotypes from our cases and 1000 Genomes Project phase 3 samples (25). The PCA results showed that our 200 cases were mostly close to the East Asian (EAS) samples, as expected confirming the ethnicity of the cases used in this study (**Figure S1**). Since MODY was a monogenic disease, we assessed the previously reported pathogenic gene of MODY in our cases. Intriguingly, we found that the variants of MODY genes were usually accompanied by rarity and functional impact in suspected MODY cases, while other type diabetes cases were not observed.

Based on the significant difference in the clinical phenotype among the three diabetic groups, we also performed GWAS analysis. FPG, FPGC-peptide, and diabetic groups were selected to investigate the genotypic and phenotypic relationships. As displayed in **Figure 2E**, several significant sites (*P*< 10 × 10^–7^, Fisher’s exact test) were found in all three association results, suggesting that those sites were highly correlated with diabetes (**Table S1**). Enrichment analysis was performed and confirmed that those genes are relevant to diabetes (**Tables S2, S3**).

### Potential Pathogenic Variants in Suspected Maturity-Onset Diabetes of the Young Cases

**Figure 3:**
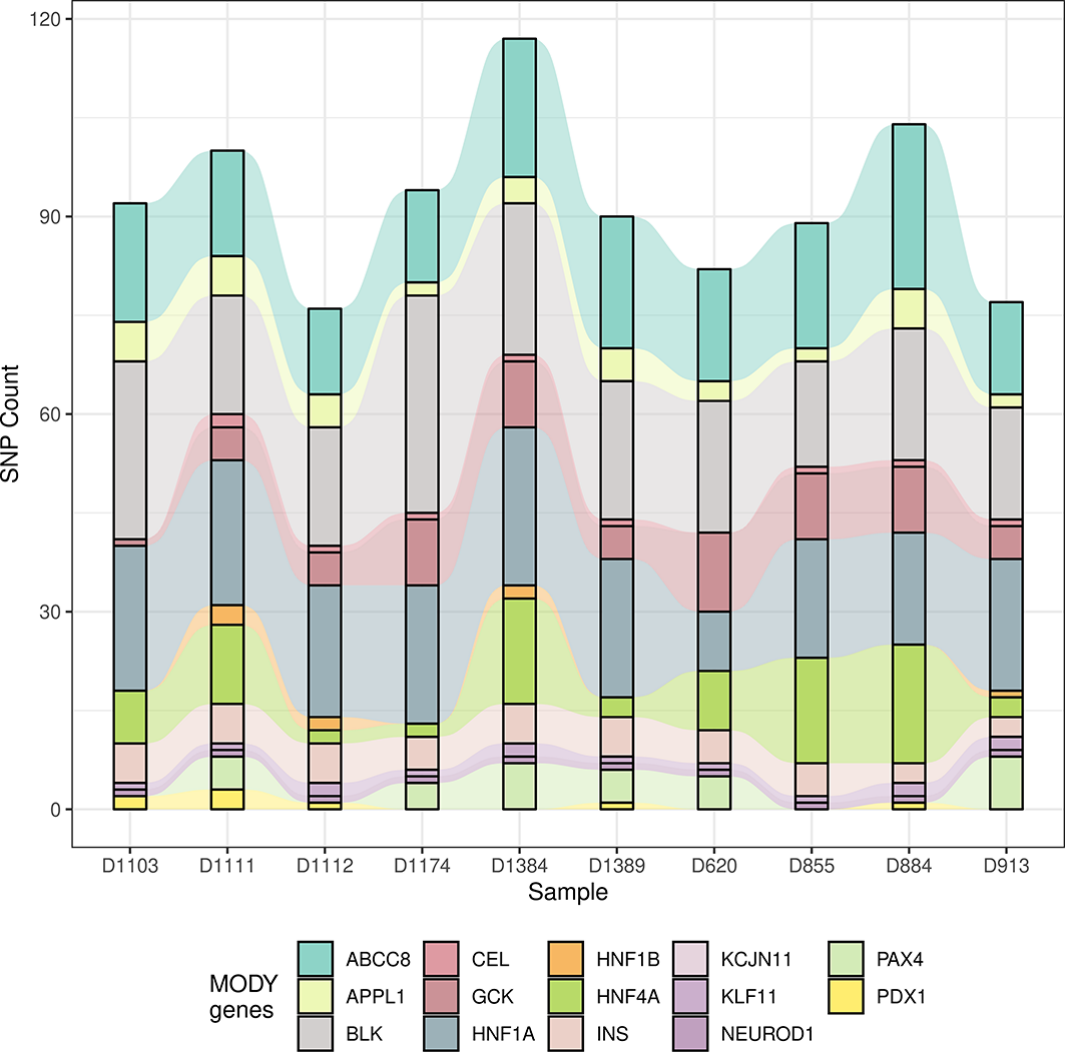
The count of mutation in 14 MODY genes. The alluvial diagram drawed by the count of mutation in 14 MODY genes. X axis indicates the name subjects and Y axis indicates the count of mutation. Different colors represent different genes.

Since the allele frequency and population prevalence of MODY were lower than those in T2DM, burden analysis of rare variants across MODY and MODY-X genes was performed. Gene-based burden test was more efficient and justified for identifying associated monogenic traits than GWAS, as a single variant might show negative result due to the low frequency and the heterogeneity of pathogenic genes (26). Based on previous reports, 14 specific MODY type genes and 40 MODY-X genes were collected for the burden test. From **Figure 3**, we found that the mutations in *HNF1A*, *ABCC8*, and *BLK* were more numerous than the others among 14 MODY genes. **Table 2** presents the mutation count of MODY and MODY-X genes in 10 suspected subjects; however, the percentage of non-synonymous mutations in MODY-X genes was higher than that of MODY genes. This implied that the prevalence of MODY in Northern China may be caused by other pathogenic genes, since the 14 types of MODY were first and widely reported among populations in Europe and America.

**Table 2:** Variation count of MODY-related genes.

	MODY	MODY non synonymous	MODY-X	MODY-X non synonymous
D620	82	1(1.22%)	35	3(8.57%)
D855	89	4(4.49%)	45	5(11.11%)
D884	104	4(3.85%)	38	4(10.53%)
D913	77	2(2.6%)	31	2(6.45%)
D1103	92	2(2.17%)	22	5(22.73%)
D1111	100	5(5%)	53	9(16.98%)
D1112	76	5(6.58%)	30	7(23.33%)
D1174	94	2(2.13%)	32	3(9.38%)
D1384	117	4(3.42%)	44	7(15.91%)
D1389	90	4(4.44%)	33	3(9.09%)

To deduce the pathogenesis of MODY in Northern China with MODY and MODY-X genes, comprehensive analyses were performed to evaluate the potential sites in those genes. Sites with allele frequencies >0.01 in the 1000 Genome Project, gnomAD-ALL and gnomAD-EAS databases were removed, and burden test was applied with RVTESTS (27) on the remaining mutations between three diabetic groups. Six genes (*PAX4, FoxA3, Nr5a2, Hnf4a, Ada, Foxm1*) reached the significance level (*P* < 0.1, adjusted by Bonferroni method) for the burden test of association (**Table S4**). However, only the sites in *ADA* and *FOXM1* annotated with non-synonymous mutations, which could potentially impact the function of proteins. Thus, several single nucleotide variants of those genes were prioritized in the subjects as potential candidates for MODY.

### Maturity-Onset Diabetes of the Young*-X Gene: FOXM1 Induced* Maturity-Onset Diabetes of the Young

**Figure 4:**
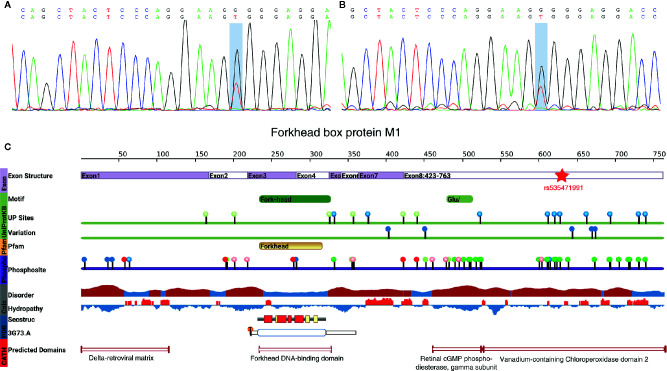
rs535471991 identified in *FOXM1*. **(A, B)** Results of Sanger sequencing of rs535471991 in *FOXM1* of the subjects. The highlighted position indicates c.T1895G and the results showed the mutation on complementary strand. **(C)** The vertical color bar on the left side indicates data provenance, the top part in lavender color indicates the genomic exon structure; the green part indicates the motif and variation information from UniProtKB, UP sites shows the Uniprot annotated amino acid modifications and Variation shows non-genetic variation from ExPASy and dbSNP; the oranges part indicates the domain information from Pfam; purple part indicates the phosphorylation annotation from PhosphoSite; the grey part indicates the disorder (red: potentially disordered region, blue: probably ordered region) and hydropathy (red: hydrophobic, blue: hydrophilic) information; the blue part indicates the annotation from PDB, Secstruc shows the secondary structure and 3G73.A shows the structure from PDB; the red part indicates homology models predicted by CATH.

As the single-variant association test of these three potential candidates did not show the association with MODY sufficiently, to address whether those variants in the genes that showed a positive result from the burden test were responsible for the pathogenesis of MODY, the impact of each mutation was thoroughly researched using clinical, population or functional databases. Fortunately, there were two pedigreed subjects among the 10 suspected cases, which was only detected in the father and the son. However, we did not find any pathogenic variant in 14 MODY genes, then extend out our sight to MODY-X genes which were collected from OMIM (Online Mendelian Inheritance in Man) annotation related with diabetes. Finally, rs535471991 was revealed as a heterozygous missense mutation (NM_202002; c.T1895G) in the coding region of *FOXM1*. This variant was then verified by Sanger sequencing (**Figures 4A, B**, **Figure S2**).

This mutation leads to an alteration in the amino acid sequence (p.Val632Gly). The allele frequency in the 1000 Genomes, Exome Aggregation Consortium (ExAC) (28) and Genome Aggregation Database (gnomAD) (29) was 2.00×10^–5^, 9.48×10^–5^ and 1.80×10^–5^ respectively, but it was not found in the Exome Sequencing Project (ESP) (30). The pathogenic prediction of SIFT, MutationTaster (31), CADD (32), and gerp++ indicated that this mutation was a deleterious mutation. Furthermore, we found that the mutation was located in vanadium-containing chloroperoxidase domain 2, which was annotated by a protein structure classification database (CATH) (33) (**Figure 4C**). That particular domain played a major role in phosphorylation, and the mutation existed exactly among a series of phosphorylation sites. A single nucleotide polymorphism at codon 1895, leading to the substitution of valine (Val) for glycine (Gly) in FOXM1, implicated the stability of the protein. The alteration caused the loss of isopropyl group and decreased the stability of FOXM1 (34). Furthermore, the hydrophobic state also changed from hydrophobicity to hydrophilicity, which played an important role in cell cycle and insulin signaling pathway, especially in pancreatic cell proliferation (35). These findings extrapolate that rs535471991 may behave as a potentially pathogenic variant in *FOXM1*.

## Discussion

Two hundred diagnosed diabatic subjects were analyzed from northern China. However, multiple susceptibility genes involved in diabetes, particularly T1DM or T2DM, presented a distinguishing feature of polygenic inheritance and differed from MODY or neonatal diabetes mellitus (NDM), since WES could provide an accurate molecular diagnosis for monogenic disease.

Clinically, we found that BMI, FPG, C-peptide, and HbA_1_ *c* had a significant difference between suspected MODY patients and T2DM, while no difference was observed with T1DM. MODY patients usually do not associate with obesity, which is consistent with previous reports. It is interesting to note that FPG and HbA_1_ *c* were higher than T2DM and C-peptide was lower than T2DM in this study and that phenomenon also occurred in the research of Zhang (12) and Anuradha (36), despite general acceptance that patients with MODY have better glycemic control than patients with T2DM. Based on the phenotypic distinctions, GWAS and gene-based burden test were performed, however, the number of MODY patient was not large enough to obtain a convincing statistical results, which bring a limitation on this study. Besides, we use MODY group as case and T1DM and T2DM as control, some significantly differential traits might also stick out in the association results, such as BMI. Thus, we chose FPG and insulin data as covariates for association analysis to adjust the result. Fortunately, 2 MODY patients was a pair of father and son, which brought us a pedigreed information for investigating the potential pathogenic loci credibly. Since MODY-X might be the main component in China, and the relevant genes could impact the glycemic control even worse than that regulated by other diabetic genes. Therefore, the percentage of non-synonymous 14 MODY genes was sharply less than that of MODY-X genes, indirectly suggesting that the prevalence in China was distinctive, which was also comparable with previous studies (11–13).

Moreover, we also found a missence mutation in *ADA* except *FOXM1*. *ADA* is located in 20q12-q13.1 and has been reported as a MODY-associated region identified by genetic map of chromosomes (37). However, the mutation of emphADA only occurred in one suspected patient, unlike *FOXM1* without pedigreed information, and we could not verify as a pathogenic site based on a solitary case. In contrast, the mutation in *FOXM1* occurred in two paternity samples. *FOXM1* appears to be a transcription factor that regulates the expression of cell cycle proteins and is essential for proper mitotic progression (38, 39). During mitosis, cyclinA-dependent binding of CDK1 (cyclin-dependent kinase 1) affects the phosphorylation of FOXM1 within the vanadium-containing chloroperoxidase domain and then regulates the S/G2 transition (40). Though the vanadium-containing chloroperoxidase domain was annotated as a disordered region that did not have a stable three-dimensional structure but had good plasticity, the disordered region modulated the stability depending on the degree of aromaticity and phosphorylation status (41). In addition, receptor-mediated insulin signaling promotes the expression and binding between *FOXM1* and *CENPA* and *PLK1* in pancreatic β-cell to enable proliferation (35). These findings suggest that the mutation in *FOXM1* most likely affects the risk of MODY.

In summary, the study focused on MODY in the Northern China population. Based on WES analysis, we report a mutation in *FOXM1* caused MODY potentially. Furthermore, we validated the genetic findings with an alternative sequencing method and performed *in silico* analysis that suggest that rs535471991 may be relevant to the development of MODY. Therefore, elucidation of the molecular genetics of *FOXM1* is likely to result in better an understanding of the pathogenesis of MODY.

## Data Availability Statement

Raw reads of whole-exome sequencing data has been submitted to the NCBI Sequence Read Archive (SRA; http://www.ncbi.nlm.nih.gov/sra/) under accession number SRP227138.

## Ethics Statement

The study was approved by the ethical committee of The Shijiazhuang Second Hospital, Shijiazhuang, China, and all the patients provided their written informed consent to participate in this study.

## Author Contributions

LZ, CL, and ZZ designed the experiment. LZ analyzed the data and wrote the manuscript. YL, QH, CL, and YX collected the samples. WZ tidied up the clinical information. NL, ZZ, and RR constructed the sequencing library. All authors contributed to the article and approved the submitted version.

## Funding

We thank the patients and the stuff in The Shijiazhuang Second Hospital in this research. This work was supported by the National Natural Science Foundation of China (81400884), Key Research and Development Program Projects in Hebei Province (20190156), and Key Research and Development Program Self-Financing Projects in Hebei Province (172777120).

## Conflict of Interest

The authors declare that the research was conducted in the absence of any commercial or financial relationships that could be construed as a potential conflict of interest.
