# Supplementary material for: Identification of Maturity-Onset Diabetes of the Young Caused by Mutation in FOXM1 via Whole-Exome Sequencing in Northern China
Source: Front Endocrinol (Lausanne). 2021 Feb 9;11:534362. doi: 10.3389/fendo.2020.534362 (PMC7900535; doi:10.3389/fendo.2020.534362)
Supplement: Supplementary file 1 [file DataSheet_1.pdf]

Identification of maturity onset diabetes of the young caused by mutation in *FOXM1* via whole-exome sequencing in Northern China

Liang Zhong[1,2,3], Zenyi Zhao[1,2], Qingshan Hu[1,2], Yang Li[1,2,3], Weili Zhao[1,2,3], Chuang Li[1,2], Yunqiang Xu[1], Ruijuan Rong[1,2,3], Jing Zhang[1,2,3], Zifeng Zhang[1,2,3], Nan Li[1,2,3], Zanchao Liu[1,2,3,*],

[1] The Shijiazhuang Second Hospital, Shijiazhuang, Hebei, China

[2] Hebei Provincial Key Laboratory of Basic Medicine for Diabetes, Shijiazhuang, Hebei, China

[3] Shijiazhuang Technology Innovation Center of Precision Medicine for Diabetes, Shijiazhuang, Hebei, China

Supplementary Table 1. Pairwise comparison between diabetic groups

	Group1	***Group2***	***p-Value***	***Adjusted p value***	***Significance***
Age	MODY	T1DM	0.00725823	0.0073	**
Age	MODY	T2DM	1.49E-06	4.50E-06	****
Age	T1DM	T2DM	0.000194237	0.00039	***
BMI	MODY	T1DM	0.2	0.22	ns
BMI	MODY	T2DM	0.00756331	0.023	**
BMI	T1DM	T2DM	0.109961397	0.22	ns
FPG	MODY	T1DM	0.406344451	0.41	ns
FPG	MODY	T2DM	0.00868066	0.017	**
FPG	T1DM	T2DM	0.001240885	0.0037	**
FPGC-peptide	MODY	T1DM	0.574835652	0.57	ns
FPGC-peptide	MODY	T2DM	0.000220456	0.00044	***
FPGC-peptide	T1DM	T2DM	1.28E-05	3.90E-05	****
HbA1c	MODY	T1DM	0.06022889	0.12	ns
HbA1c	MODY	T2DM	0.000239988	0.00072	***
HbA1c	T1DM	T2DM	0.688947849	0.69	ns
Insulin	MODY	T1DM	0.969851098	0.97	ns
Insulin	MODY	T2DM	0.063225047	0.13	ns
Insulin	T1DM	T2DM	0.002002185	0.006	**
Hepatic Fat Content	T1DM	T2DM	0.203	0.2	ns
Cholesterol	MODY	T1DM	0.49214177	0.98	ns
Cholesterol	MODY	T2DM	0.216746626	0.65	ns
Cholesterol	T1DM	T2DM	0.981847741	0.98	ns
Serum creatinine	MODY	T1DM	0.53932819	1	ns
Serum creatinine	MODY	T2DM	0.918421945	1	ns
Serum creatinine	T1DM	T2DM	0.23998686	0.72	ns
Triglyceride	MODY	T1DM	0.197685565	0.4	ns
Triglyceride	MODY	T2DM	0.683405314	0.68	ns
Triglyceride	T1DM	T2DM	0.062083274	0.19	ns
HDL	MODY	T1DM	0.920108282	1	ns
HDL	MODY	T2DM	0.666186795	1	ns
HDL	T1DM	T2DM	0.897402908	1	ns
LDL	MODY	T1DM	0.569700822	1	ns
LDL	MODY	T2DM	0.304233578	0.91	ns
LDL	T1DM	T2DM	0.755838935	1	ns

[1] The pairwise comparision was performed by Wilcoxon test. The P value was adjusted by Benjamini & Hochberg(FDR) method.

[2] 'ns': p > 0.05 ; '*': p <= 0.05 ; '**': p <= 0.01 ; '***': p <= 0.001 ; '****': p <= 0.0001

Supplementary Table 2. Variation count in 14 type MODY gene

	D620	D855	D884	D913	D1103	D1111	D1112	D1174	D1384	D1389
Hnf4A	9	16	18	3	8	12	2	2	16	3
Gck	12	10	10	5	1	5	5	10	10	5
Hnf1A	9	18	17	20	22	22	20	21	24	21
Pdx1	0	0	1	0	2	3	1	0	0	1
Hnf1B	0	0	0	1	0	3	2	0	2	0
Neurod1	1	1	1	1	1	1	1	1	1	1
Klf11	1	1	2	2	1	1	2	1	2	1
Cel	0	1	1	1	0	2	1	1	1	1
Pax4	5	0	0	8	0	5	0	4	7	5
Ins	5	5	3	3	6	6	6	5	6	6
Blk	20	16	20	17	27	18	18	33	23	21
Abcc8	17	19	25	14	18	16	13	14	21	20
Kcjn11	0	0	0	0	0	0	0	0	0	0
Appl1	3	2	6	2	6	6	5	2	4	5

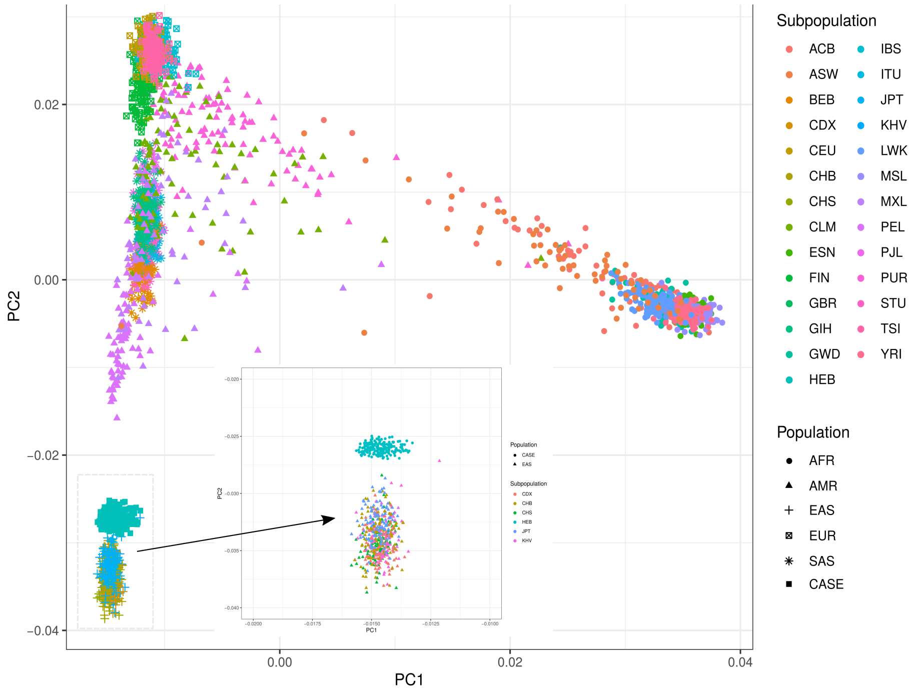

Supplementary Figure 1. PCA plot 200 subjects and 1000 Genome Project The combined PCA analysis of 200 subjects and all samples from 1000 Genome Project. The main population (AFR: African; AMR: American; EAS: East Asian; EUR: European; SAS: South Asian) and 200 diabetic subjects (CASE) were group by different shapes. 27 Sub-population were in group by different colors. The detailed PCA analysis of EAS samples and the 200 subjects was also displayed in the bottom of figure.

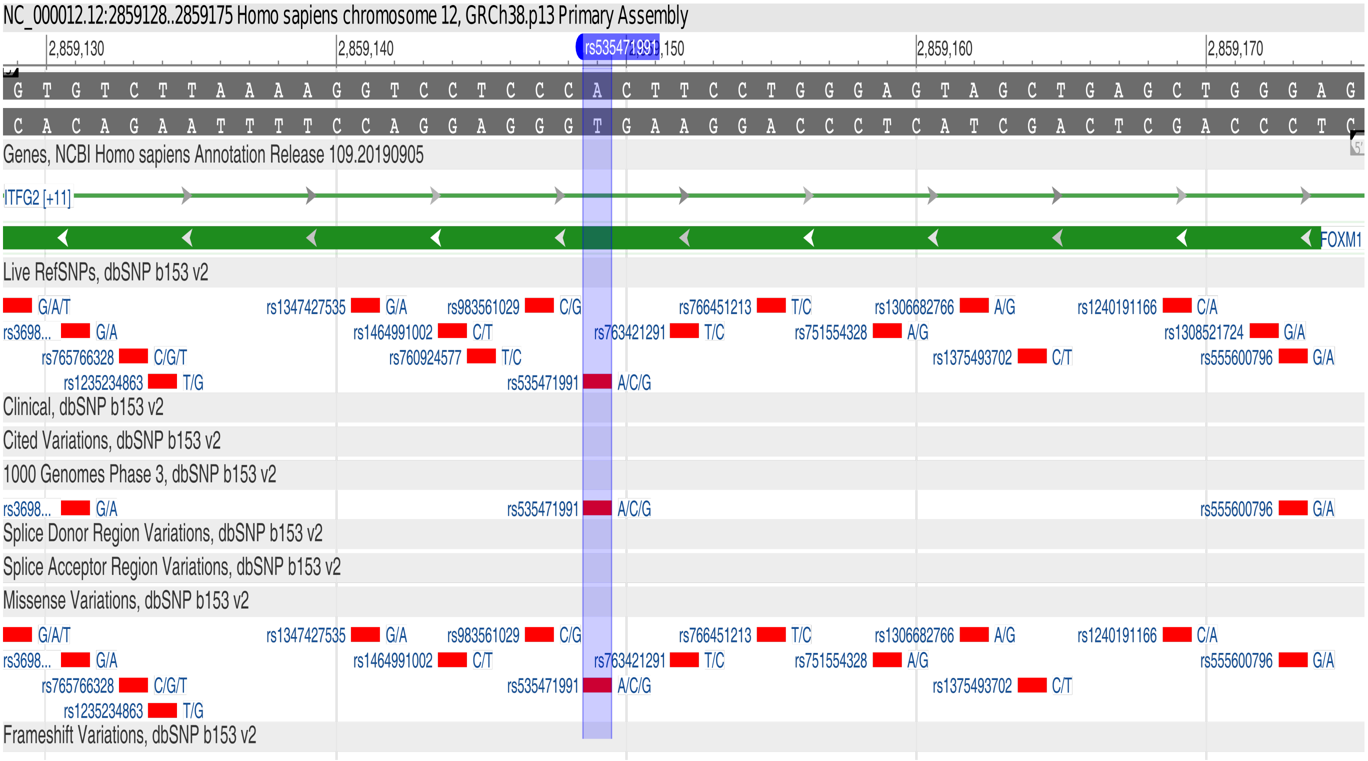

Supplementary Figure 2. rs535471991 annotated in variation databases The genomic information and the variation annotation from various databases of rs535471991.
